# Comorbidity and Non-prosthetic Inpatient Rehabilitation Outcomes After Dysvascular Lower Extremity Amputation

**DOI:** 10.33137/cpoj.v3i1.33916

**Published:** 2020-05-16

**Authors:** M.G Marquez, M Kowgier, W.S Journeay

**Affiliations:** 1 Department of Anatomy and Cell Biology, McGill University, Montreal, Canada.; 2 Dalla Lana School of Public Health, University of Toronto, Toronto, Canada.; 3 Providence Healthcare – Unity Health Toronto, Toronto, ON, Canada.; 4 Division of Physical Medicine and Rehabilitation, Department of Medicine, University of Toronto, Toronto, Canada.

**Keywords:** Amputation, Comorbidity, Dysvascular, Inpatient Rehabilitation, Charlson Comorbidity Index, Functional Independence Measure, Diabetes, Limb Loss, Rehabilitation, Amputee

## Abstract

**BACKGROUND:**

Dysvascular amputations arising from peripheral vascular disease and/or diabetes are common. Patients who undergo amputation often have additional comorbidities that may impact their recovery after surgery. Many individuals undergo post-operative inpatient rehabilitation to improve their non-prosthetic functional independence. Thus far, our characterization of comorbidity in this population and how it is associated with non-prosthetic inpatient functional recovery remains relatively unexplored.

**OBJECTIVE:**

The objective of this study was to describe comorbidities, using the Charlson Comorbidity Index (CCI), and to examine associations between comorbidity and functional outcomes in a cohort of patients with dysvascular limb loss undergoing non-prosthetic inpatient rehabilitation.

**METHODOLOGY:**

A retrospective cohort design was used to analyze a group of 143 patients with unilateral, dysvascular limb loss who were admitted to inpatient rehabilitation. Age, sex, amputation level, amputation side, length of stay (LOS), time since surgery, Functional Independence Measure (FIM) scores (Total and Motor at admission and discharge), and CCI scores were collected.

**FINDINGS:**

The data showed that neither total or specific comorbidities were associated with functional outcomes or LOS in this cohort and rehabilitation model. Multivariate analysis demonstrated an inverse relationship with age and FIM scores, where increased age was associated with lower Total and Motor FIM at admission and discharge. Comorbidities were not associated with functional outcomes. Dementia was negatively associated with FIM scores, however this requires more study given the low number of patients with dementia in this cohort.

**CONCLUSION:**

These data suggest that regardless of burden of comorbidity or specific comorbidities that patients with dysvascular limb loss may derive similar functional benefit from post-operative non-prosthetic inpatient rehabilitation.

## INTRODUCTION

The primary risk factors for lower extremity amputation (LEA) are diabetes and peripheral arterial disease along with associated dysvascular complications.^1–4^ When combined, peripheral arterial disease and diabetes are associated with greater than 80% of LEA in Canada ^4,5^ and recent population-based research by Hussain et al.,^6^ demonstrated that diabetes-related amputations are on the rise. Moreover, patients with dysvascular limb loss often carry a burden of comorbidity including cognitive impairment and heart failure among others which can potentially further impact recovery and function in hospitalized patients after amputation.^7,8^

After a LEA, patients may be discharged to inpatient rehabilitation, specialized nursing facilities, or directly home, with the destination contingent on factors such as the patient’s age, level of amputation, and family support^9–12^ as well as the location and availability of rehabilitation facilities. Inpatient rehabilitation is particularly beneficial as it correlates to fewer additional amputations, reduced mortality, a greater probability of receiving a prosthesis, and improved medical stability.^10,13^ Regardless of one’s prosthetic candidacy, patients undergoing amputation have a number of post-operative rehabilitation needs including transfer training, wheelchair skills and contracture prevention. A patient’s stay in inpatient rehabilitation and medical status after surgery may be impacted not only by the amputation, but also by other comorbidities they may have.^12^ To date, few studies have examined comorbidity in patients with dysvascular limb loss and/its association with functional outcomes and length of stay in the non-prosthetic inpatient rehabilitation setting.

A measure that can be used to quantify comorbidity is the Charlson Comorbidity Index (CCI).^14,15^ In the classical chart review version of the CCI, it is split into individual conditions, each assigned a weighted score of either 1, 2, 3, or 6. A higher total score indicates a greater burden of comorbidities and a higher risk of mortality in hospitalized patients. The Functional Independence Measure (FIM) is a standardized indicator of functional progress in the inpatient rehabilitation setting and such data can be readily obtained from rehabilitation hospital data reporting systems.^16,17^ Given its common use in inpatient rehabilitation settings, the FIM can be used as a clinical marker to reflect progression in self care, transfers and wheelchair independence needed before non-prosthetic discharge.

The majority of prior studies have catalogued common comorbidities without using an established index or they employed various measures of functional outcome outside of the inpatient non-prosthetic setting. For example, Melchiorre et al.^8^ have investigated the relationship between comorbidities and rehabilitation for patients with LEA. However, their work focused on amputations of etiology that were both traumatic and vascular. They used a modified CCI in order to reflect their study sample, and looked at correlations with length of stay (LOS) and FIM scores. Chopra et al.^18^ also studied the relationship between comorbidities and functional outcomes in patients with lower extremity amputation. Their study did not focus on inpatient rehabilitation, as most of their cohort were discharged to skilled nursing facilities. They measured function by observing patient independence with activities of daily living (ADL) and ambulation. They did not use the CCI, but tallied several specific comorbidities. Vogel et al.,^7^ looked at the impact of amputation and comorbidities using the CCI in nursing home residents with LEA. Due to the elderly cohort and their disposition in a nursing home, the treatment provided was more residential rather than focused predominantly on post-operative, non-prosthetic rehabilitation. They also measured function with performance of ADLs. Cheng et al. found no association of comorbidity with unplanned discharge or functional gains however they utilized specific medical predictors rather than an index such as the CCI.^19^ The role of comorbidity is increasingly an important area of study as even in patients with dyvascular limb loss under the age of 65 years old there exists a high burden of comorbidity.^20^

While there has been previous work concerning the role of comorbidity on prosthetic rehabilitation outcomes, there has not been a more comprehensive look into the distinct components of the CCI and its association with inpatient non-prosthetic rehabilitation outcomes including the FIM and LOS. Thus, with increasing rates of amputation related to diabetes^6^ and a greater number of patients needing rehabilitation,^12^ this remains a relevant topic to explore. Therefore, the purpose of this study was to describe comorbidities, using the CCI, and to examine associations between comorbidity, functional outcomes and LOS in a cohort of patients with dysvascular lower limb loss undergoing non-prosthetic inpatient rehabilitation.

## METHODOLOGY

This was a retrospective cohort study and was approved by the Research Ethics Board of Providence Healthcare and closed by the Unity Health Toronto Research Ethics Board. All patients with a LEA that were discharged from our rehabilitation hospital between January 1, 2014 and March 30, 2018 were identified and their medical records were reviewed. Inclusion criteria for the study consisted of those with a recent unilateral, transfemoral (TF) or transtibial (TT) amputation. Only those amputations with a dysvascular or diabetic cause were included, and those due to trauma, cancer or other reasons were excluded. Those receiving hemodialysis were also excluded from this study as data from this group was used in a separate comparative study. Inclusion and exclusion criteria were developed to establish a uniform data set of the most common reason for admission to post-amputation rehabilitation (dyvascular amputation). Patients who met inclusion criteria but had an incomplete data set were excluded.

All data retrieved from medical records came from both physical charts and electronic files utilized by Health Information Management at the hospital. The rehabilitation model at this institution involved post-operative interdisciplinary rehabilitation including physiotherapy, occupational therapy, nursing, wound care and physiatry consultation. The focus of rehabilitation for these patients was non-prosthetic rehabilitation only which includes but is not limited to; wound care, standing tolerance, contracture prevention, transfers and wheelchair skills. Patients were discharged home after non-prosthetic rehabilitation and then revisited regarding prosthetic candidacy and gait training at a later date. Data that was extracted from the medical records included age, sex, amputation level, amputation side, surgery date, LOS in inpatient rehabilitation, FIM scores at admission and discharge,^16,17^ and CCI scores.^14,15^ The authors are aware that the CCI was initially used as an epidemiological tool to predict mortality in patients admitted to hospital. However, we have selected it as a standardized method in which to catalogue comorbidities.

Each patient was reviewed using the CCI and assigned points for the individual conditions, then given a total score. These scores were based on information present upon their admission and any past medical history that was documented in the chart. The time since surgery was also recorded by calculating the number of days between the surgery date and the admission date to inpatient rehabilitation. LOS in rehabilitation was calculated from admission date to discharge date. Total FIM, and total motor FIM information was retrieved from admission and discharge data. We included motor FIM because in the non-prosthetic phase of rehabilitation, the motor FIM scores would reflect acquisition of independence with transfers and wheelchair mobility as this study did not examine prosthetic gait outcomes.

### Statistical Methods

Continuous variables were summarized by observed means with standard deviation (SD) and categorical variables were summarized by frequency counts (percentages). Univariate and multivariate linear regression analyses were used to investigate the effect of comorbidities on each of the outcomes of Total and Motor FIM at both admission and discharge as well as LOS. Multiple regression analysis adjusted for clinically relevant variables including age and sex as well as any comorbidities showing statistical association (p < 0.05) in univariate analysis. Data was analyzed using the R statistical software (version 3.5.1).

## RESULTS

All patients admitted with a diagnosis of LEA from January 1, 2014 to March 30, 2018 were identified by our medical records team for a total of 382 charts. Three patients were excluded due to death prior to discharge. Four patients were excluded due to incomplete admission to discharge data sets. Twenty-five patients were excluded due to hemodialysis. Two hundred and seven charts were excluded by not meeting inclusion criteria such as: etiology of amputation (i.e. not dysvascular), had bilateral amputations, or were not TT or TF level amputations (i.e. only forefoot or toe amputation), or were not admitted postoperatively but rather for other reasons such as gait training or other medical conditions. There was a total of 143 patients who met inclusion criteria and were analyzed (Table 1).

**Table 1:** Cohort characteristics and Charlson Comorbidity Index.

		Cohort n = 143 (%)
**Age (years)**		67.7 (SD 11.1)
**Sex**	M	95 (66)
	F	48 (34)
**Amputation**	Transfemoral	59 (41)
**Level**	Transtibial	84 (59)
**Amputation**	Left	69 (48)
**Side**	Right	74 (52)
**Length of stay in rehabilitation (LOS)**		33.9 (SD 18.6)
**Time since surgery to admission (days)**		15.2 (SD 13.8)
**FIM scores**	Overall Total Admission	72.6 (SD 14.4)
	Overall Total Discharge	97.5 (SD 14.3)
	Motor Total Admission	42.7 (SD 12.0)
	Motor Total Discharge	66.9 (SD 11.4)
	Efficiency	0.9 (SD 0.5)
**Charlson Total**		4.7 (SD 1.7)
**Charlson Comorbidity** **Index items**	Peripheral Vascular Disease	136 (95)
	High Blood Pressure	124 (87)
	Diabetes Mellitus	78 (55)
	Skin Ulcer	60 (42)
	Chronic Obstructive Pulmonary Disease	31 (22)
	Myocardial Infarction	27 (19)
	Cerebrovascular Accident	19 (13)
	Diabetes Mellitus-End Organ	19 (13)
	Congestive Heart Failure	16 (11)
	Depression	13 (9)
	Cancer / Malignancy	9 (6)
	Warfarin	9 (6)
	Peptic Ulcer Disease	6 (4)
	Dementia	6 (4)
	Rheumatic Disease	4 (3)
	Renal Disease	3 (2)
	Mild Liver Disease	1 (1)
	Mod-Sev Liver Disease	2 (1)
	Metastatic cancer	1 (1)
	Human Immunodeficiency Virus	0 (0)
	Hemiplegia	0 (0)

The majority of the cohort was male (66%) and the mean age was 68 years old. Most of the cohort had a TT level amputation (59%). Ninety-five percent of the cohort had peripheral vascular disease (PVD), 68% had diabetes mellitus (DM), 87% had hypertension (HBP), and 42% had a skin ulcer. Table 1 presents further descriptive data, along with the distribution of the rest of the individual comorbidity scores.

Total CCI or dichotomized CCI (>6 or <6 score) were not associated with FIM scores or LOS. The individual comorbidities that were shown to have an association with lower overall total FIM scores at admission, after univariate analysis, were cerebrovascular disease (CVA) (Beta=-8.09, CI=[(-14.94) - (-1.24)], P=0.022), dementia (Beta=-23.46, CI=[(-34.63) - (-12.30)], P<0.001), and HBP (Beta=-9.46, CI=[(-16.26) - (-2.65)], P=0.007). At discharge, CVA (Beta= -7.82, CI=[(-14.63) - (-1.01)], P=0.026), dementia (Beta= -28.85, CI=[(-39.57) - (-18.12)], P<0.001), and HBP (Beta= -7.78, CI=[(-14.59) - (-0.96)], P=0.027) also showed an association with lower overall total FIM scores. Age was also associated with a lower overall total FIM scores, at both admission (Beta=-0.58, CI=[(-0.78) - (-0.39)], P<0.001) and discharge (Beta=-0.47, CI=[(-0.67) - (-0.28)], P=0.000). Sex showed an association with motor FIM scores at admission such that females had lower scores (Beta=-4.36, CI=[(-8.46) - (-0.25)], P=0.040). At admission, the comorbidities associated with lower motor FIM scores were dementia (Beta=-16.39, CI=[(-25.84) - (-6.95)], P=0.001) and HBP (Beta=-6.41, CI=[(-12.11) - (-0.70)], P 0.029). Dementia was the only comorbidity to show an association with lower motor FIM scores at discharge (Beta -19.42, CI=[(-28.18) - (-10.66)], P=0.000). Age was negatively associated with motor FIM scores at admission (Beta -0.45, CI=[ (-0.61) - (-0.29)], P<0.001) and discharge (Beta -0.33, CI=[ (-0.49) - (-0.17)], P<0.001). The remaining univariate analyses are presented in Table 2.

**Table 2:** Univariate analysis, FIM Total, FIM Motor and LOS *P<0.05

		**FIM Total Admission**			**FIM Total Discharge**			**FIM Motor Admission**			**FIM Motor Discharge**			**Length of Stay (LOS)**		
		Beta	CI	P-value	Beta	CI	P-value	Beta	CI	P-value	Beta	CI	P-value	Beta	CI	P-value
Sex (Female vs Male)		-3.51	[(-8.49) - (1.47)]	0.170	-3.06	[(-8.01) - (1.90)]	0.229	-4.36	[(-8.46) - (-0.25)]	0.040*	-3.86	[(-7.77) - (0.05)]	0.055	-1.04	[(-7.53) - (5.44)]	0.753
Amp Side (Left vs Right)		-1.47	[(-6.20) - (3.27)]	0.545	-1.89	[(-6.58) - (2.81)]	0.433	-0.13	[(-4.07) - (3.81)]	0.948	-1.25	[(-4.99) - (2.49)]	0.513	3.60	[(-2.50) - (9.70)]	0.249
Amp Level (TF vs TT)		-3.06	[(-7.84) - (1.73)]	0.213	-1.92	[(-6.69) - (2.85)]	0.431	-3.05	[(-7.02) - (0.92)]	0.134	-2.78	[(-6.55) - (0.99)]	0.151	-2.19	[(-8.39) - (4.02)]	0.491
Age		-0.58	[(-0.78) - (-0.39)]	0*	-0.47	[(-0.67) - (-0.28)]	0*	-0.45	[(-0.61) - (-0.29)]	0*	-0.33	[(-0.49) - (-0.17)]	0*	0.24	[(-0.04) - (0.51)]	0.092
Charlson Total Score		-1.28	[(-2.66) - (0.09)]	0.070	-1.08	[(-2.45) - (0.30)]	0.126	-1.13	[(-2.28) - (0.01)]	0.054	-0.93	[(-2.02) - (0.16)]	0.096	0.76	[(-1.04) - (2.56)]	0.410
Charlson (>= 6 vs <6)		-3.64	[(-8.81) - (1.53)]	0.169	-2.72	[(-7.87) - (2.43)]	0.302	-3.80	[(-8.08) - (0.48)]	0.084	-3.11	[(-7.19) - (0.96)]	0.136	0.33	[(-6.39) - (7.06)]	0.923
Charlson items	Myocardial infarction	0.58	[(-5.48) - (6.63)]	0.852	1.93	[(-4.07) - (7.94)]	0.529	-0.09	[(-5.13) - (4.94)]	0.971	1.22	[(-3.55) - (5.99)]	0.618	-0.19	[(-8.02) - (7.63)]	0.962
	Congestive Heart Failure	-2.98	[(-10.48) - (4.52)]	0.438	-1.94	[(-9.39) - (5.52)]	0.612	-4.74	[(-10.93) - (1.46)]	0.136	-3.87	[(-9.77) - (2.03)]	0.201	5.22	[(-4.45) - (14.90)]	0.292
	Peripheral vascular disease	-3.53	[(-14.49) - (7.44)]	0.529	-4.16	[(-15.05) - (6.72)]	0.454	0.44	[(-8.68) - (9.57)]	0.924	-0.67	[(-9.33) - (8.00)]	0.880	3.01	[(-11.17) - (17.19)]	0.678
	Cerebrovascular accident	-8.09	[(-14.94) - (-1.24)]	0.022*	-7.82	[(-14.63) - (-1.01)]	0.026*	-5.25	[(-10.98) - (0.49)]	0.075	-4.66	[(-10.12) - (0.79)]	0.096	5.08	[(-3.90) - (14.06)]	0.270
	COPD	0.08	[(-5.66) - (5.83)]	0.977	2.37	[(-3.33) - (8.06)]	0.417	-1.31	[(-6.09) - (3.46)]	0.591	1.27	[(-3.26) - (5.81)]	0.582	3.31	[(-4.10) - (10.72)]	0.383
	Diabetes - end organ	3.32	[(-3.63) - (10.28)]	0.351	-0.24	[(-7.17) - (6.69)]	0.947	1.98	[(-3.82) - (7.77)]	0.505	-0.78	[(-6.28) - (4.73)]	0.783	1.19	[(-7.83) - (10.21)]	0.796
	Skin Ulcer	-3.80	[(-15.60) - (8.00)]	0.529	1.08	[(-10.66) - (12.81)]	0.858	-6.13	[(-15.90) - (3.64)]	0.221	-0.46	[(-9.78) - (8.87)]	0.924	5.19	[(-10.06) - (20.44)]	0.506
	Metastatic Cancer	-0.33	[(-10.09) - (9.42)]	0.947	4.48	[(-5.18) - (14.14)]	0.365	0.91	[(-7.20) - (9.01)]	0.827	3.62	[(-4.05) - (11.30)]	0.356	-1.75	[(-14.35) - (10.86)]	0.786
	Dementia	-23.46	[(-34.63) - (-12.30)]	0*	-28.85	[(-39.57) - (-18.12)]	0*	-16.39	[(-25.84) - (-6.95)]	0.001*	-19.42	[(-28.18) - (-10.66)]	0*	-3.51	[(-18.77) - (-11.75)]	0.653
	Rheumatic diseas	-5.03	[(-19.38) - (9.31)]	0.493	2.35	[(-11.91) - (16.61)]	0.748	-8.44	[(-20.30) - (3.42)]	0.165	-1.22	[(-12.56) - (10.12)]	0.833	5.54	[(-13.00) - (24.09)]	0.559
	High blood presssure	-9.46	[(-16.26) - (-2.65)]	0.007*	-7.78	[(-14.59) - (-0.96)]	0.027*	-6.41	[(-12.11) - (-0.70)]	0.029*	-4.08	[(-9.55) - (1.39)]	0.146	8.40	[(-0.52) - (17.31)]	0.067
	Skin Ulcer	-3.06	[(-7.84) - (1.71)]	0.211	-2.64	[(-7.39) - (2.10)]	0.277	-3.26	[(-7.21) - (0.70)]	0.109	-2.88	[(-6.64) - (0.88)]	0.136	2.74	[(-3.45) - (8.93)]	0.387
	Depression	1.66	[(-6.58) - (9.90)]	0.693	3.55	[(-4.62) - (11.71)]	0.396	0.58	[(-6.27) - (7.43)]	0.869	2.10	[(-4.40) - (8.60)]	0.527	-1.96	[(-12.61) - (8.68)]	0.719
	Warfarin	-0.92	[(-10.68) - (8.83)]	0.853	-1.45	[(-11.13) - (8.24)]	0.770	1.38	[(-6.73) - (9.49)]	0.739	-0.53	[(-8.23) - (7.17)]	0.894	-7.08	[(-19.64) - (5.47)]	0.271

The factors that showed an association after the univariate analysis were then adjusted using multivariate analysis. For overall total FIM scores at admission and discharge, dementia (Admission P=0.007; Discharge P<0.001) and age (Admission P<0.001; Discharge P=0.001) were shown to be inversely associated with overall total FIM scores at admission after adjusting for confounders. Age was negatively associated with lower motor FIM scores at admission (Estimate=-0.41, SD=0.09, P<0.001). Being female was also inversely associated with lower motor FIM scores at admission after adjusting for other confounders (Estimate=-3.94, SD=1.91, P=0.042). Dementia showed a negative association with motor FIM at discharge (Estimate=–14.27, SD=4.61, P=0.002). Age also showed an association with poorer motor FIM scores at discharge (Estimate=-0.27, SD=0.08, P=0.002). Table 3 includes remaining data from multivariate analysis.

**Table 3:** Multivariate analysis, FIM Total, FIM Motor and LOS. *P<0.05

	**FIM Total Admission**			**FIM Total Discharge**			**FIM Motor Admission**			**FIM Motor Discharge**			**Length of Stay (LOS)**		
	Estimate (SE)	t value	P-value	Estimate (SE)	t value	P-value	Estimate (SE)	t value	P-value	Estimate (SE)	t value	P-value	Estimate (SE)	t value	P-value
Intercept	112.38 (6.70)	16.77	0.000	126.31 (6.84)	18.45	0.000	74.67 (5.75)	12.98	0.000	87.82 (5.68)	15.47	0.000	10.59 (9.82)	1.08	0.283
Sex (F vs M)	-2.63 (2.23)	-1.18	0.241	-1.35 (2.28)	-0.59	0.555	-3.94 (1.91)	-2.06	0.042	-2.82 (1.89)	-1.49	0.137	-0.24 (3.27)	-0.07	0.942
Age	-0.5 (0.1)	-5.03	<0.001*	-0.35 (0.1)	-3.47	0.001*	-0.41 (0.09)	-4.81	<0.001*	-0.27 (0.08)	-3.18	0.002*	0.25 (0.15)	1.72	0.087
CVA	-4.39 (3.08)	-1.42	0.157	-4.35 (3.15)	-1.38	0.170	-2.75 (2.65)	-1.04	0.301	-2.5 (2.61)	-0.96	0.340	2.23 (4.52)	0.49	0.622
Dementia	-15.01 (5.44)	-2.76	0.007*	-22.73 (5.56)	-4.09	<0.001*	-8.84 (4.67)	-1.89	0.061	-14.27 (4.61)	-3.10	0.002*	-6.07 (7.97)	-0.76	0.448
High blood pressure	-3.49 (3.13)	-1.12	0.266	-2.93 (3.2)	-0.92	0.361	-1.74 (2.69)	-0.65	0.520	-0.55 (2.65)	-0.21	0.835	5.34 (4.59)	1.16	0.246

## DISCUSSION

The aim of this study was to describe comorbidities and the association with functional outcomes and length of stay in a cohort of patients with recent dysvascular limb loss undergoing inpatient non-prosthetic rehabilitation. There are three main findings from this study including **1**: We identified the distribution of comorbidities using the CCI in a cohort of patients with dysvascular limb loss admitted to inpatient rehabilitation, **2**: Age and dementia were two main factors associated with inpatient Total and Motor FIM scores. **3**: None of the individual comorbidities included in the CCI were associated with LOS in this cohort undergoing non-prosthetic rehabilitation. Many patients with limb loss entering inpatient rehabilitation programs have a burden of comorbidity in addition to their amputation. Identification of which factors possibly hinder these patients during inpatient rehabilitation may assist in supporting these often complex and frail patients after amputation surgery.

The distribution of demographic items in this dysvascular cohort was similar to prior studies. This study focused on patients with unilateral, transfemoral and transtibial limb loss with similar proportions of amputation level as well as average age in comparison with prior published work.^21,22^ A study conducted by Taylor et al. reviewing patients with a major LEA showed comparable ratios of patients with PVD and DM.^23^ Of note, the three most frequent comorbidities were PVD, hypertension and diabetes which may underscore the need for medical management and secondary prevention in this population.

This study is unique in that it examines the distribution of the CCI items in patients with dysvascular limb loss and the association of each of these with inpatient non-prosthetic functional outcomes and length of stay. A study done by Arneja et al.^24^ examined functional outcomes between patients with LEA on dialysis and those not on dialysis. In their study only discharge FIM scores were included, while our study contained both admission and discharge FIM. Additionally, their study examined various comorbidities but did not use an established index such as the CCI. Overall, in our study the total CCI score did not show strong associations with functional outcomes in this cohort after multivariate analysis. FIM changes and scores in this cohort generally reflect acquisition of independence with transfers and wheelchair mobility as this study did not examine prosthetic gait outcomes. A study conducted by Stewart et al.^25^ provided evidence that patients with chronic conditions, such as cardiac and pulmonary disease, which are captured in the CCI, do have an impact on function. The discrepancy between these findings and the absence of associations from our data could be explained by the nature of the patients in our study. These patients are medically complex and admitted to rehabilitation for only a short period of time to address non-prosthetic independence, so their progression may not be as evident with the outcome measures studied. Conversely, the lack of differences attributed to specific comorbidities suggests that patients undergoing dysvascular amputation should still be offered non-prosthetic rehabilitation and can benefit from postoperative rehabilitation services regardless of comorbidity burden. Moreover, there was no association of comorbidity with LOS suggesting that despite multiple comorbidities these patients can achieve a non-prosthetic functional level sufficient for discharge in a similar amount of time while admitted to inpatient rehabilitation.

While the total CCI score was not associated with functional outcomes, our results show that dementia had a significant association with FIM scores. Dementia was found to have an inverse association with overall total FIM, at both admission and discharge, and motor FIM, at discharge. A limitation in our study was that there were only six patients who fit the inclusion criteria and had dementia upon admission. This finding is in accordance with past studies that have also demonstrated the relationship of cognitive impairment with poor functional outcomes after amputation.^23,26–29^ Given, the very low number of patients with dementia in this data set, this association must be interpreted with caution as additional research with a larger sample size would be required to draw broader conclusions.

Age was another factor that was found to have an association with total and motor FIM at both admission and discharge, with advanced age resulting in lower FIM scores. This result is reasonable on account that it has been shown that patients are more likely to accumulate more medical conditions as they age.^30^ There are also additional studies that support the notion that advanced age is associated with poorer functional outcomes in patients with limb loss.^27,31^ However, another report by Chopra et al. did not indicate an association between greater age and poorer ambulatory rates, which they attributed to their cohort size.^18^

### Limitations

There was an association with dementia and functional outcomes however these patients represented a very small portion of the cohort and therefore future work should be directed to larger cohorts to better understand this association. Furthermore, this study examined only the post-operative and non-prosthetic component of hospitalized rehabilitation patients with recent limb loss. While burden of comorbidity and specific comorbidities did not show associations with functional outcomes in this cohort it raises a number of additional points. While, one would hypothesize that a greater burden of comorbidity such as cardiovascular disease would impact ambulatory function, this cohort admitted for non-prosthetic rehabilitation was not impacted. This suggests that patients referred post-operatively after amputation who may never be prosthetic candidates may still benefit from inpatient rehabilitation to recover from surgery and restore independence prior to discharge. The CCI reflects specific medical comorbidities however other factors may also play a role in rehabilitation after limb loss including the condition of the contralateral limb, visual impairments, delayed wound healing and mental health status, which could be explored in future studies. An important comorbidity which may disproportionately impact function and may not be fully reflected in the CCI is that of end-stage renal disease in patients receiving hemodialysis. In patients living with limb loss who also receive dialysis, the mortality and functional outcomes are much poorer than those with dysvascular amputation and no hemodialysis.^21,32^ In order to better address this question in a non-prosthetic inpatient rehabilitation setting additional comparative studies (dyvascular amputation vs dysvascular plus dialysis) are needed. Furthermore, this cohort represents one post-amputation care model in Canada and therefore the results may not be directly generalized to other forms of rehabilitation which can vary locally, nationally and internationally.

## CONCLUSION

In summary, we report the distribution of comorbidities in a cohort of patients with dysvascular limb loss using the CCI. There was an association with dementia and functional outcomes as represented by the FIM, however larger sample sizes will be needed to better explore this association. Age did show negative associations with FIM scores. There were no associations of comorbidity with inpatient rehabilitation length of stay. Finally, given that there were no significant associations between total or specific comorbidities and functional outcomes and LOS in this cohort, medically complex patients with limb loss may still derive benefit from post-operative, non-prosthetic inpatient rehabilitation to restore independence prior to discharge from hospital.

## DECLARATION OF CONFLICTING INTERESTS

The authors have no conflicts of interest to declare.

## AUTHOR CONTRIBUTION

**Michelle G. Marquez**:

Completed data collection, data interpretation, literature review and manuscript writing.

**Matthew Kowgier**:

Assisted in study design, led statistical analysis and contributed to manuscript development.

**W. Shane Journeay**:

Conceived the study and design, data interpretation and manuscript writing.

## SOURCES OF SUPPORT

Michelle G. Marquez received a Providence Healthcare student research stipend.

## ETHICAL APPROVAL

This was a retrospective cohort study and was approved by the Research Ethics Board of Providence Healthcare and closed by the Unity Health Toronto Research Ethics Board.

## References

[1] Schofield CJ, Libby G, Brennan GM, MacAlpine RR, Morris AD, Leese GP. Mortality and hospitalization in patients after amputation: a comparison between patients with and without diabetes. Diabetes Care. 2006; 29:2252-6. DOI:10.2337/dc06-0926 17003302

[2] Ziegler-Graham K, MacKenzie EJ, Ephraim PL, Travison TG, Brookmeyer R. Estimating the prevalence of limb loss in the United States: 2005 to 2050. Arch Phys Med Rehabil. 2008; 89(3):422-9. DOI: 10.1016/j.apmr.2007.11.005 18295618

[3] Kayssi A, de Mestral C, Forbes TL, Roche-Nagle G. A Canadian population-based description of the indications for lower-extremity amputations and outcomes. Can J Surg. 2016; 59:99-106. DOI: 10.1503/cjs.013115 27007090 PMC4814278

[4] Imam B, Miller WC, Finlayson HC, Eng JJ, Jarus T. Incidence of lower limb amputation in Canada. Can J Public Health. 2017; 108:e374-e380. DOI: 10.17269/cjph.108.6093 29120308 PMC6975214

[5] Kayssi A, de Mestral C, Forbes TL, Roche-Nagle G. Predictors of hospital readmissions after lower extremity amputations in Canada. J Vasc Surg. 2016; 63:688-95. DOI:10.1016/j.jvs.2015.09.017 26610648

[6] Hussain MA, Al-Omran M, Salata K, Sivaswamy A, Forbes TL, Sattar N, et al. Population-based secular trends in lower-extremity amputation for diabtes and peripheral vascular disease. CMAJ. 2019; 191:E955-61. DOI: 10.1503/cmaj.190134 31481423 PMC6721859

[7] Vogel TR, Petroski GF, Kruse RL. Impact of amputation level and comorbidities on functional status of nursing home residents after lower extremity amputation. J Vasc Surg. 2014; 59:1323-1330. DOI: 10.1016/j.jvs.2013.11.076 24406089 PMC4004653

[8] Melchiorre PJ, Findley T, Boda W. Functional outcome and comorbidity indexes in the rehabilitation of the traumatic versus the vascular unilateral lower limb amputee. Am J Phys Med Rehabil. 1996; 75:9-14. 8645444 10.1097/00002060-199601000-00004

[9] Lavery LA, Van Houtum WH, Armstrong DG. Institutionalization following diabetes-related lower extremity amputation. Am J Med. 1997; 103:383-388. DOI:10.1016/S0002-9343(97)00163-0 9375706

[10] Dillingham TR, Pezzin LE. Rehabilitation setting and associated mortality and medical stability among persons with amputations. Arch Phys Med Rehabil. 2008; 89:1038-1045. DOI:10.1016/j.apmr.2007.11.034 18503797

[11] Dillingham TR, Yacub JN, Pezzin LE. Determinants of postacute care discharge destination after dysvascular lower limb amputation. PM R. 2011; 3:336-344. DOI:10.1016/j.pmrj.2010.12.019 21497320 PMC5391794

[12] Kayssi A, Dilkas S, Dance DL, de Mestral C, Forbes TL, Roche-Nagle G. Rehabilitation trends after lower extremity amputations in Canada. PM R. 2016; 9:494-501. DOI: 10.1016/j.pmrj.2016.09.009 27664402

[13] Stineman MG, Kwong PL, Kurichi JE, Prvu-Bettger JA, Vogel WB, Maislin G, et al. The effectiveness of inpatient rehabilitation in the acute postoperative phase of care after transtibial or transfemoral amputation: Study of an integrated health care delivery system. Arch Phys Med Rehabil. 2008; 89:1863-72. DOI: 10.1016/j.apmr.2008.03.013 18929014 PMC2880880

[14] Charlson ME, Pompei P, Ales KL, MacKenzie CR. A new method of classifying prognostic comorbidity in longitudinal studies: development and validation. J Chronic Dis. 1987; 40:373-383. DOI:10.1016/0021-9681(87)90171-8 3558716

[15] Charlson ME, Charlson RE, Briggs W, Hollenberg J. Can disease management target patients most likely to generate high costs? The impact of comorbidity. J Gen Intern Med. 2007; 22(4):464-469. DOI:10.1007/s11606-007-0130-7 17372794 PMC1829434

[16] Hamilton BB, Laughlin JA, Fiedler RC, Granger CV. Interrater reliability of the 7-level functional independence measure (FIM). Scand J Rehabil Med. 1994; 26:115-119. 7801060

[17] Keith RA, Granger CV, Hamilton BB, Sherwin FA. The functional independence measure: A new tool for rehabilitation. Adv Clin Rehabil. 1987; 1:6-18. PMID: 3503663

[18] Chopra A, Azarbal AF, Jung E, Abraham CZ, Liem TK, Landry GJ, et al. Ambulation and functional outcome after major lower extremity amputation. J Vasc Surg. 2018; 67:1521-1529. DOI: 10.1016/j.jvs.2017.10.051 29502998

[19] Cheng R, Smith SR, Kalpakjian CZ. Comorbidity has no impact on unplanned discharge or functional gains in persons with dysvascular amputation. J Rehabil Med. 2019; 51(5):369-375. DOI: 10.2340/16501977-2554. 30964543

[20] Mayo AL, Cimino SR, Hitzig SL. A depiction of rehabilitation patients 65 years and younger with dysvascular lower extremity amputation. Can Prosthet Orthot J. 2019; 2(1):1 DOI: 10.33137/cpoj.v2i2.33505 PMC10443480 37614808

[21] Nehler MR, Coll JR, Hiatt WR, Regensteiner JG, Schnickel GT, Klenke WA, et al. Functional outcome in a contemporary series of major lower extremity amputations. J Vasc Surg. 2003; 38(1):7-14. DOI: 10.1016/s0741-5214(03)00092-2 12844082

[22] Batten H, Kuys S, McPhail S, Varghese P, Mandrusiak A. Are people with lower limb amputation changing? A seven-year analysis of patient characteristics at admission to inpatient rehabilitation and at discharge. Disabil Rehabil. 2019; 41(26):3203-3209. DOI:10.1080/09638288.2018.1492033 30182758

[23] Taylor SM, Kalbaugh CA, Blackhurst DW, Hamontree SE, Cull DL, Messich HS, et al. Preoperative clinical factors predict postoperative functional outcomes after major lower limb amputation: an analysis of 553 consecutive patients. J Vasc Surg. 2005; 42: 227-235. DOI: 10.1016/j.jvs.2005.04.015 16102618

[24] Arneja AS, Tamiji J, Hiebert BM, Tappia PS, Galimova L. Functional outcomes of patients with amputation receiving chronic dialysis for end-stage renal disease. Am J Phys Med Rehabil. 2015; 94: 257-268. DOI: 10.1097/PHM.0000000000000259 25785920

[25] Stewart AL, Greenfield S, Hays RD, Wells K, Rogers WH, Berry SD, et al. Functional status and well-being of patients with chronic conditions. Results from the Medical Outcomes Study. JAMA. 1989; 262(7): 907-913. DOI:10.1001/jama.1989.03430070055030 2754790

[26] Heinemann AW, Linacre JM, Wright BD, Hamilton BB, Granger C. Prediction of rehabilitation outcomes with disability measures. Arch Phys Med Rehabil. 1994; 75: 133-143. DOI: 10.1016/0003-9993(94)90385-9 8311668

[27] Schoppen T, Boonstra A, Groothoff JW, de Vries J, Goeken LN, Eisma WH. Physical, mental, and social predictors of functional outcome in unilateral lower-limb amputees. Arch Phys Med Rehabil. 2003; 84: 803-811. DOI:10.1016/S0003-9993(02)04952-3 12808530

[28] Frengopoulos C, Burley J, Viana R, Payne MW, Hunter SW. Association between Montreal Cognitive Assessment Scores and measures of functional mobility in lower extremity amputees after inpatient rehabilitation. Arch Phys Med Rehabil. 2017; 98: 450-455. DOI:10.1016/j.apmr.2016.06.012 27422347

[29] Williams RM, Turner AP, Green M, Norvell DC, Henderson AW, Hakimi KN, et al. Relationship between cognition and functional outcomes after dysvascular lower extremity amputation: a prospective study. Am J Phys Med Rehabil. 2015; 94(9): 707-17. DOI: 10.1097/PHM.0000000000000235 25357146 PMC7518634

[30] Johnson VJ, Kondziela S, Gottschalk F. Pre and post-amputation mobility of trans-tibial amputees: correlation to medical problems, age and mortality. Prosthet Orthot Int. 1995; 19: 159-164. DOI:10.3109/03093649509167999 8927527

[31] Covinsky KE, Palmer RM, Fortinsky RH, Counsell SR, Stewart AL, Kresevic D, et al. Loss of independence in activities of daily living in older adults hospitalized with medical illnesses: increased vulnerability with age. J Am Geriatr Soc. 2003; 51: 451-458. DOI:10.1046/j.1532-5415.2003.51152.x 12657063

[32] Serizawa F, Sasaki S, Fujishima S, Akamatsu D, Goto H, Amada N. Mortality rates and walking ability transition after lower limb major amputation in hemodialysis patients. J Vasc Surg. 2016; 64(4): 1018-25. DOI: 10.1016/j.jvs.2016.03.452 27189770

